# Cyclic precipitation variation on the western Loess Plateau of China during the past four centuries

**DOI:** 10.1038/srep06381

**Published:** 2014-09-16

**Authors:** Liangcheng Tan, Zhisheng An, Chih-An Huh, Yanjun Cai, Chuan-Chou Shen, Liang-Jian Shiau, Libin Yan, Hai Cheng, R. Lawrence Edwards

**Affiliations:** 1 State Key Laboratory of Loess and Quaternary Geology, Institute of Earth Environment, Chinese Academy of Sciences, Xi'an 710075, China; 2 Institute of Earth Sciences, Academia Sinica, Taipei 11529, Taiwan; 3 High-precision Mass Spectrometry and Environment Change Laboratory (HISPEC), Department of Geosciences, National Taiwan University, Taipei 106, Taiwan; 4 Institute of Global Environmental Change, Xi'an Jiaotong University, Xi'an 710049, China; 5 Department of Earth Sciences, University of Minnesota, Minneapolis 55455, USA

## Abstract

Precipitation variation on the Loess Plateau (LP) of China is not only important for rain-fed agriculture in this environmentally sensitive region, but also critical for the water and life securities over the whole Yellow River basin. Here we reconstruct high resolution precipitation variation on the western LP during the past 370 years by using two replicated, annually-laminated stalagmites. Spatial analysis suggests that the reconstruction can be also representative for the whole LP region. The precipitation variations show a significant quasi-50 year periodicity during the last 370 years, and have an important role in determining the runoff of the middle Yellow River. The main factor controlling the decadal scale variations and long-term trend in precipitation over this region is southerly water vapour transport associated with the Asian summer monsoon. The Pacific Decadal Oscillation is also an important influence on precipitation variation in this region, as it can affect the East Asian summer monsoon and the West Pacific Subtropical High.

The Loess Plateau (LP) is one of the most important cradles of Chinese civilization^1^, and today more than 100 million people live in this region. Located on the northern limit of the East Asian summer monsoon (EASM), this environmentally sensitive region depends largely on summer monsoon precipitation to sustain its agriculture. In addition, as the main sediment source and water-catchment area of the Yellow River, the LP is the most important water source for northern China^2^. Precipitation variations on the LP are critical for the water and life securities over the whole Yellow River basin^3^,^4^. Widely distributed loess/paleosol sequences in the LP have revealed the East Asian monsoon variability over tectonic, orbital and millennial timescales^5^,^6^,^7^,^8^. However, the loess/paleosol sequences cannot record high-frequency climate variations because of their sedimentation and diagenetic processes. Despite some paleoprecipitation reconstructions^9^,^10^,^11^, the characteristic and mechanism of decadal scale precipitation variation on the LP, especially the western LP, during historical times are still poorly understood. Moreover, the relationship between extreme decadal scale rainfall/drought events on the LP and the water security of the Yellow River remain unclear.

Wuya Cave (33°49′14″ N, 105°25′35″ E, 1370 m a.s.l.) is located on the southwestern margin of LP, Gansu Province, China (Fig. 1). Regional climate is semiarid, with annual precipitation of 470 mm and annual temperature of 14.9°C (Supplementary Fig. S1). In summer, the summer monsoon brings warm humid air and causes substantial monsoon rainfall at the site, with ~80% of the annual rainfall falling between May and September (data from Wudu meteorological station, 60 km southwest of Wayu Cave). During winter, the Siberian–Mongolian High and westerly winds maintain cold and dry conditions^12^.

Two columnar stalagmites (Supplementary Fig. S2), with lengths of 63 mm for WY27 and 84.5 mm for WY33, were collected inside the cave about 500 m from the entrance in March 2011. Both stalagmites were receiving drip water from the ceiling at the time of collection. The polished sections of the stalagmites show continuous, clear laminae with alternations from a dark, compact layer (DCL) to a white, porous layer (WPL). The visible white layers are opaque under transmission light microscope, but luminescent under mercury light source UV reflected light (Supplementary Fig. S3), suggesting they are mainly composed of organic substances^13^. As suggested by observations in other caves^14^,^15^, annual flush of organic substances from the soil during monsoon rain seasons may form these laminar divisions.

## Results

Lamina counting results suggest that there are 370 ± 15 and 262 ± 10 (±4% error applied to the lamina counts) DCL-WPL couplets in WY27 and WY33, respectively. High detrital ^232^Th levels of 7–28 × 10^3^ ppt result in large dating uncertainty of 113–874 years for ^230^Th dates (Supplementary Table S1) and hinder precise age models.

To further constrain the chronology, we use ^210^Pb dating. The ^210^Pb dating results show exponential decrease of excess ^210^Pb activities with growth depth in the two stalagmites (Supplementary Figs. S4, S5 and Table S2), indicating the upper sections of WY27 and WY33 are younger than ~130 years^16^,^17^. The average growth rate calculated from the depth profile of excess ^210^Pb for the top 23.5 mm of WY27 (0.171 mm/yr) is in good agreement with that estimated by lamina counting (0.156 mm/yr). The growth rate for the top 39 mm of WY33 determined by ^210^Pb (0.289 mm/yr) is slightly lower than that calculated by lamina counting (0.361 mm/yr), which may be caused by the high sampling width (3–5 mm) and the errors of ^210^Pb dating (<10%) and lamina counting (±4%). The comparison suggests the DCL-WPL couplets in both stalagmites are annual. According to the layer counting chronologies, WY27 grew from 1641 to 2010 CE, and WY33 from 1749 to 2010 CE (Supplementary Fig. S6).

**Figure 2:**
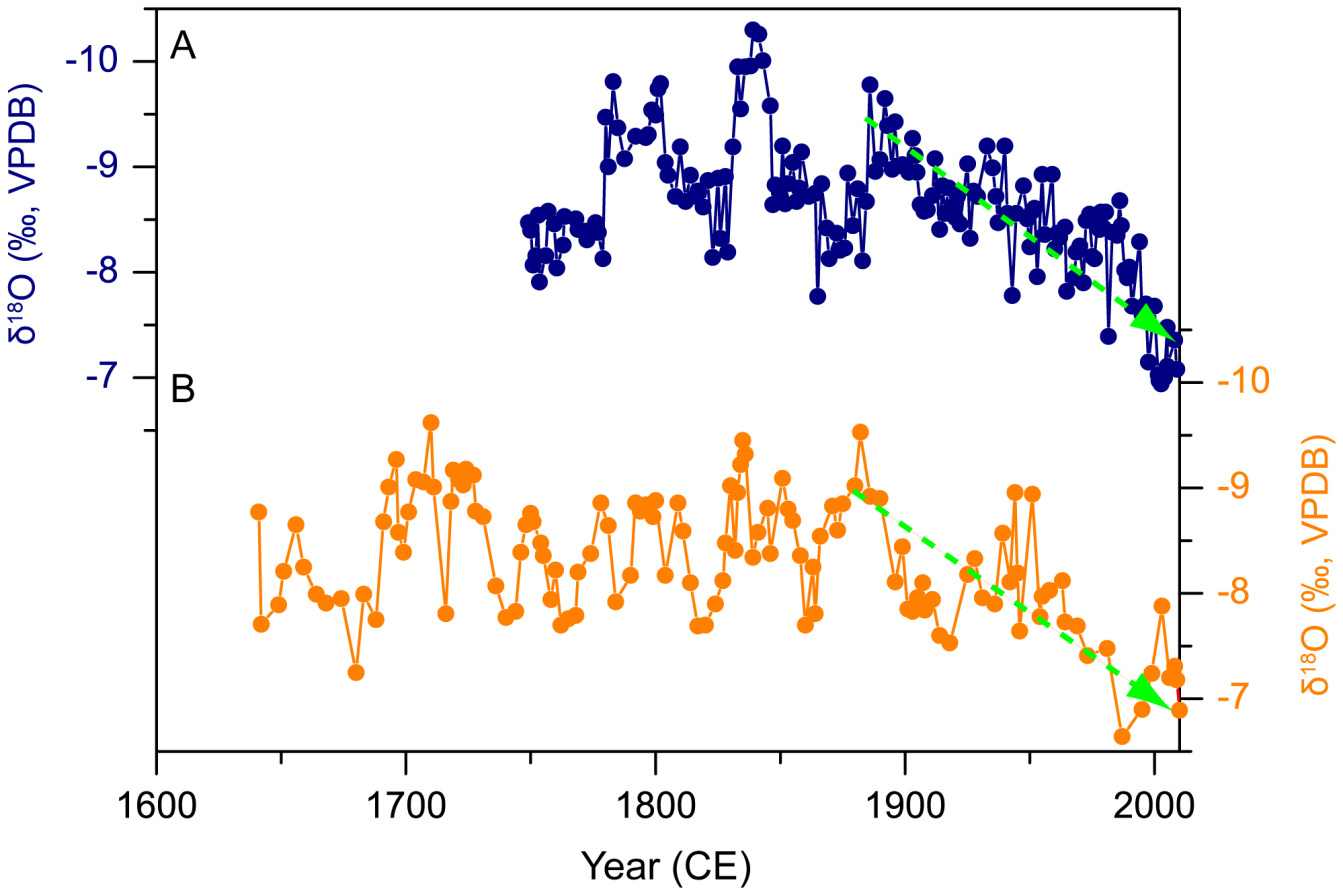
The stalagmite δ^18^O series of (A) WY33 and (B) WY27. The green, broken lines with arrows in both series indicate linear trends toward more enriched values since the end of the 19^th^ century. A minor difference of 0.5‰ was observed for the contemporaneous δ^18^O segments of the WY27 and WY33 records, which may be attributable to slightly prior calcite precipitation in different water flow paths above the stalagmites^11^,^60^.

Stalagmites WY27 and WY33 show similar decadal scale δ^18^O variations during the contemporaneous period of 1749–2010 CE (Fig. 2), but WY33 has a higher average resolution (1.6 yrs) than WY27 (2.9 yrs, Supplementary Table S3). The replicated δ^18^O records lend further support to the laminal chronologies. A “Hendy test”^18^ on three growth layers of WY33 and two growth layers of WY27 indicates the δ^18^O remain constant along a single growth layer (Supplementary Fig. S7). The replicated δ^18^O records and “Hendy test” results suggest both stalagmites were deposited under oxygen isotopic equilibrium conditions^18^,^19^.

Stalagmite δ^18^O variation can be affected by different factors, including temperature, rainfall amount, moisture recycling and circulation, change in the precipitation-to-evaporation ratio, and changes in the moisture source and transport pathway^20^,^21^,^22^,^23^. Previous studies^11^,^12^ suggested there was an anti-correlation between speleothem δ^18^O and rainfall amount in this monsoonal front region during the last 1800 years on decadal to centennial scales. Recently, by combining simulation records and paleo-moisture records, Liu et al.^24^ suggest speleothem δ^18^O records in northern China can represent the EASM intensity and local monsoon rainfall, with negative δ^18^O records representing enhanced monsoon intensity and monsoon rainfall in northern China.

The comparison between our annually-laminated stalagmite δ^18^O and the annual rainfall (mainly contributed by monsoonal rainfall) records from the nearest meteorological station confirm a robust anti-phase relationship between our stalagmite δ^18^O and local rainfall amount (*r* = −0.44, *p* < 0.01) on both decadal scale and long term trend during the observed period of 1951–2009 CE (Supplementary Fig. S8). Good coherence of stalagmite δ^18^O and meteorological record supports the robustness of this laminal chronology and an isotopic equilibrium statement during deposition.

The δ^13^C records of the two stalagmites show similar variations with their δ^18^O records on annual- to decadal- scales (Supplementary Fig. S9), although their long term trends differ (Supplementary Figs. S10, S11). The correlation coefficient for the detrended δ^13^C (Δ^13^C) and δ^18^O records of WY27 (Δ^18^O) is *r* = 0.42 (*p* < 0.01), and is *r* = 0.52 (*p* < 0.01) for WY33. During wetter conditions, the decreased residence time of the seepage water may cause less bedrock to be dissolved, resulting in lower stalagmite δ^13^C values. In addition, a wet climate favors vegetation growth and biological productivity, which may also contribute to the relatively lower δ^13^C values on a decadal scale. In contrast, the increased residence time of the seepage water during drier condition may allow more dissolution of the bedrock, resulting in the heavier δ^13^C values^25^. Decreased precipitation may also reduce vegetation cover, and favor prior carbonate precipitation in the unsaturated zone above the cave, and enhanced degassing of CO_2_ within the cave^26^,^27^, also resulting in higher δ^13^C values. Thus, the co-variations of the Δ^13^C and Δ^18^O time series lead further support to the anti-phase relationship between our stalagmite δ^18^O and local rainfall amount on annual- to decadal- timescales.

Spatial analysis shows significant positive correlations between precipitation changes around Wuya Cave area and the LP, especially the western LP during the period 1950–2011 CE (Supplementary Fig. S12). Therefore, we interpret our stalagmite δ^18^O record as a reliable indicator of precipitation changes on the western LP and even the whole LP region, with higher stalagmite δ^18^O values representing lower precipitation and *vice versa*.

**Figure 3:**
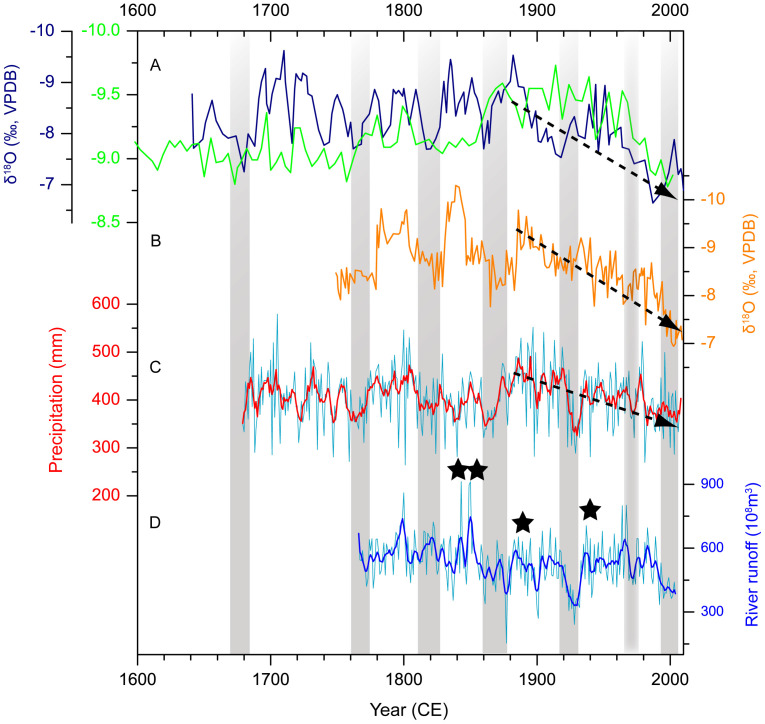
Comparison of stalagmite δ^18^O records with tree-ring record on the western LP and river runoff record of the middle Yellow River during the past 370 years. (A) WY27 δ^18^O record from Wuya Cave (dark blue) and HY3 δ^18^O record from Huangye Cave^11^ (Green); (B) WY33 δ^18^O record from Wuya Cave; (C) Tree-ring based annual rainfall reconstruction from Mt. Xinglong^10^ (the red line is smoothed with a 5-year running mean); (D) Historical river runoff reconstruction for the middle Yellow River from Sanmenxia hydrological station^37^ (the blue line is smoothed with a 5-year running mean). The shaded gray bars indicate correlations of various proxies to seven drought events on the western LP. The black, broken lines with arrows indicate a decreasing trend of precipitation on the western LP since the end of the 19^th^ century. The stars in panel D indicate four large outburst events of the Yellow River in 1841–1843, 1855, 1887 and 1938 CE^33^,^38^.

Our stalagmite δ^18^O records show good coherence with the tree-ring based annual rainfall reconstruction from Mt. Xinglong^10^ (Fig. 3), and drought reconstruction from Mt. Guiqing^9^ on the western LP. They are also consistent with a previously published relative low resolution stalagmite δ^18^O record from the Huangye Cave^11^ in this area (Fig. 3). Based on the reconstructions, a series of decadal-scale drought events on the western LP during the last 370 years can be identified: 1670s–1680s, 1760s–early 1770s, 1810s–1820s, 1860s–1870s, late 1910s–1920s, 1970s, and late 1990s. A remarkable decreasing trend in the precipitation since the end of the 19^th^ century is shown in both speleothem time series and the tree ring record (Fig. 3). In contrast, notable negative δ^18^O peaks, which indicate humid climate, can be observed in both series during the periods of 1690–1730 CE, 1780–1800 CE, 1830–1850 CE, and 1880–1900 CE.

The identified drought intervals on the western LP during the last 370 years show a periodicity of about half a century (Fig. 3). Power spectrum analysis indicates that the most significant periodicity of the WY33 δ^18^O series is 52 years, which passes the 99% significant level (Supplementary Fig. S13), further supporting the quasi-50-year periodicity.

## Discussion

### Impacts

There were two large-scale land reclamations on the western LP during the past four centuries. The first one happened in the early 18^th^ century during the Qing Dynasty^28^,^29^, and the other one happened in the late 1930s and 1940s^30^. Both land reclamations corresponded to humid periods in our reconstruction (Fig. 3), suggesting that humid climate, in addition to the orientation of policy^28^,^29^,^30^, provided favorable conditions for agricultural developments on the western LP during these periods.

Decadal scale droughts on the western LP during historical times caused serious damage to the society in this environmental sensitive region. For example, historical documents recorded the Weihe River on the western LP dried up in the summer of 1862 CE when a serious drought occurred^31^. The drought in the late half of the 1870s was much more serious and affected a vast region from LP to North China Plain, and even from South and Southeast Asia to the Great Basin of North America^32^. Chinese local officers at that time regarded this drought as the severest one since the establishment of the Qing Dynasty (1644–1911 CE), and referred to it as “Dingwu Disaster”^33^. The drought had caused catastrophic collapse of the society in northern China. About half of the population (160–200 million) was affected, and at least 10 million people were killed by famine and pestilence caused by the severe drought^33^,^34^. The affected areas in China of the drought in the 1920s were similar to that in the 1870s^35^. Descriptions like “not even a blade of grass grows”, “river dried up”, “big cannibalism”, “terrible holocaust” were reported in the newspapers at that time^33^,^36^.

The historical river runoff changes of the middle Yellow River from Sanmenxia hydrological station during the period of 1766–2004 CE^37^ match the stalagmite-inferred precipitation data (Fig. 3). The synchroneity indicates their close relationship. For instance, when droughts occurred in the 1860s–1870s, late 1920s, 1970s, and the late 1990s on the LP, abnormally low river runoff of the middle Yellow River was observed (Fig. 3). In contrast, four megafloods of the Yellow River occurring in 1841–1843, 1855, 1887 and 1938 CE^33^,^38^ coincided with the humid intervals in this region (Fig. 3).

### Driving forces

**Figure 4:**
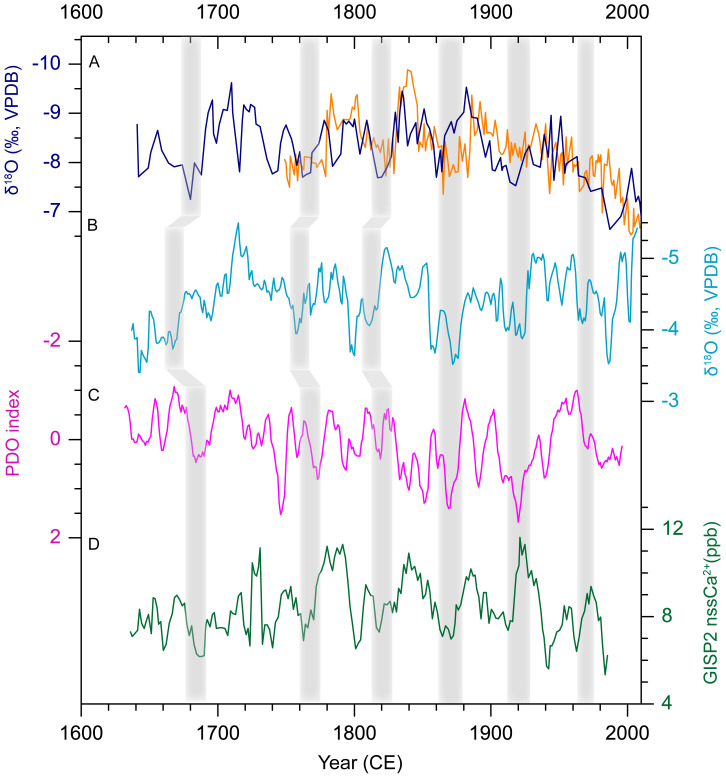
Comparisons among stalagmite δ^18^O records from Wuya Cave, Indian monsoon proxy, PDO index and Westerly proxy records. (A) WY27 (blue) and WY33 (yellow) δ^18^O records. The systematic difference of −0.5‰ between WY 33 and WY27 records was removed; (B) Stalagmite δ^18^O record from the core monsoon region of India^41^; (C) Tree-ring based PDO index^44^ (smoothed with a 5-year running mean); (D) Northern Hemisphere westerly index based on Ca^2+^ in GISP2 ice core^51^ (smoothed with a 5-year running mean). The shaded gray bars indicate drought events on the western LP and their correlation with various proxies.

The positive teleconnection between summer precipitation over northern China and Indian monsoon have been reported according to modern instrumental data^39^. A recent study^40^ suggested during the monsoonal season of the last 50 years (1961–2012CE) water vapor over the LP was transported mainly from the tropical Indian and Pacific Oceans by southerly monsoon flow, and that southerly water vapor transport controlled the interannual variability of monsoon precipitation on the LP. Our stalagmite δ^18^O records show broad similarities with the stalagmite δ^18^O record from the core monsoon region of India^41^ on decadal scale during the last 370 years (Fig. 4). The drought events recorded in our records were also observed in the Nepal Himalaya, an Indian monsoon front region, as inferred from a 223 years tree ring δ^18^O record^42^. The coincidence of the drought events in the Indian monsoon region and the western LP suggests the important influence of Indian monsoon on our study area. When the Indian summer monsoon declines, weakened southwesterly winds reduce the water vapor transport from the tropical ocean to the north, and induce a drought on the western LP. Conversely, strengthened Indian summer monsoon can enhance the southwesterly winds and the water vapor transport, resulting in increased monsoon precipitation on the western LP^43^.

The stalagmite-inferred decadal-scale droughts on the Western LP coincide with positive values of Pacific Decadal Oscillation (PDO) index (Fig. 4), i.e. warm PDO phases^44^. The correspondence is especially significant during the severe droughts in the 1860s–1870s and late 1910s–1920s. The only exception is the drought in the 1810s–1820s, which seems to correspond to a cold PDO phase in Fig. 4. However, in another two records of PDO reconstructed from the historical rainfall proxy data over eastern China^45^ and the tree ring data over Asia^46^, the PDO was in its warm phase during this drought event. It has been suggest the East Asian monsoon was weak and the Western Pacific Subtropical High (WPSH) moved southward during warm PDO phases. These conditions could result in decreased precipitation in northern China, including the western LP^39^,^47^. In contrast, the strengthened East Asian monsoon and northward shifting of the WPSH in cold PDO phases could enhance the monsoon precipitation on the western LP^39^,^47^.

The significant 52-year cycle (Supplementary Fig. S13) observed in the stalagmite δ^18^O time series is generally consistent with a 55–60 years cycle in the observed Indian monsoon rainfall^48^ and the 50–70 years cycle in the PDO index records^49^, further supporting the important influences of Indian monsoon and PDO on precipitation variations on the western LP.

Previous studies^39^,^43^,^50^ reveal a stepwise shift of the EASM and its associated migrations of monsoon air masses and rain belt were closely related to seasonal changes in the westerly upper-level jet stream and the WPSH. As the western LP is located near the northern limit of the EASM, it has been suggested the westerly jet could be an important factor for the regional precipitation by affecting the northward shift of the monsoon rain belt. By comparing Wuya stalagmite records with the reconstructed strength of the Northern Hemisphere westerly in GISP2 ice core^51^, we find concomitant changes between decadal-scale droughts on the western LP in the 20^th^ century and the strengthened westerly jet (Fig. 4). However, the opposite is true before the 20^th^ century. Therefore, the relationship between the precipitation on the western LP and the Northern Hemisphere westerly on decadal scale is still uncertain.

Modern instrumental data show a declining trend of the EASM since the end of the 19^th^ century^52^. Although the trend of the Indian summer monsoon during the past century remains debatable^53^, the observed seasonality of wind field at 850 hPa within the South Asian domain suggests a decreasing monsoon trend since 1948 CE^54^. The reduced southerly water vapor transport caused by declined Asian summer monsoon may contribute to the decreasing trend in precipitation variations on the western LP since the end of the 19^th^ century.

## Methods

We used a hand-held carbide dental drill, with a 0.9-mm diameter drill bit, to recover subsamples (50–100 mg powder) along the growth axes of WY27 and WY33 for ^230^Th dating. Seven subsamples were dated with U-series methods at the Minnesota Isotope Laboratory on an inductively coupled plasma mass spectrometer (Thermo-Finnigan ELEMENT) using procedures described in ref. 55 and ref. 56. Corrections for initial ^230^Th were made assuming an initial ^230^Th/^232^Th atomic ratio of 4.4 ± 2.2 × 10^−6^.

A total of 20 subsamples were drilled along the growth axes of the stalagmites, and were dated with ^210^Pb methods at the Institute of Earth Sciences, Academia Sinica. About 400–700 mg powders were dissolved in HCl. ^210^Pb activities were determined via ^210^Po by alpha spectrometry using ^209^Po as a yield determinant^57^. The analytical error based on counting statistics was <3%.

About 300 powdered subsamples (~50–80 μg) were drilled out by using a hand-held carbide dental drill, with a 0.3-mm diameter drill bit, at an interval of 0.5 mm along the central growth axes of the stalagmites for stable isotope analyses. All isotopic compositions were measured on an IsoPrime100 gas source stable isotope ratio mass spectrometer equipped with a MultiPrep system at the Institute of Earth Environment, Chinese Academy of Sciences. The δ^18^O values reported here are relative to the Vienna PeeDee Belemnite (VPDB) standard. Repeated measurements of one internal laboratory standard TTB1 showed that the long-term reproducibility of the δ^18^O analyses was better than ±0.06‰ (1σ).

The spectral analysis was performed using Redfit35 with parameters of the software set at values as follows: nsim = 1000, mctest = T, rhopre = −99.0, ofac = 2, n50 = 4, iwin = 1 (see ref. 58 for details).

## Author Contributions

L.T. and Z.A. directed the project; L.T. and C.C.S. designed the experiments. C.A.H. and L.J.S. conducted the ^210^Pb dating. L.T. and Y.C. performed stalagmite δ^18^O analysis and lamina counting. L.T., R.L.E. and H.C. were responsible for ^230^Th dating, and Y.L. was responsible for moisture transport analysis. All authors contributed towards preparing the manuscript.

## Supplementary Material

Supplementary Information Supplementary materials

## Figures and Tables

**Figure 1:**
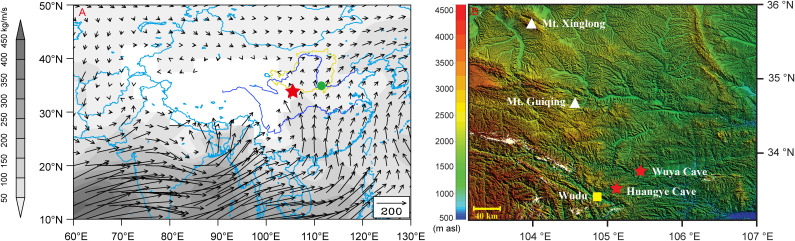
Location of Wuya cave and moisture transport pathway. (A) Rainy season (Jun.–Sep.) water vapor transport (kg m^−1^s^−1^) averaged for the period 1961–2012 at low level (700 hPa) based on the NCEP/NCAR reanalysis datasets^59^, using the Grid Analysis and Display System (GrADS). The shading shows absolute value of water vapor transport at each grid. The red star indicates Wuya Cave. The green solid circle indicates Sanmenxia hydrological station where the historical river runoff data of the Yellow River are monitored. The area enclosed in yellow line denotes the Loess Plateau; (B) An enlarged map showing the locations of Wuya Cave, Huangye Cave, Mt. Xinglong, Mt. Guiqing, and the Wudu meteorological station. Topographic GTOPO30 data are from the USGS Earth Resources Observation and Science Center.
