# Supplementary material for: Cyclic precipitation variation on the western Loess Plateau of China during the past four centuries
Source: Sci Rep. 2014 Sep 16;4:6381. doi: 10.1038/srep06381 (PMC4165273; doi:10.1038/srep06381)
Supplement: Supplementary Information — Supplementary materials [file srep06381-s1.pdf]

Supplementary Materials for

Cyclic precipitation variation on the western Loess Plateau of China during the past four centuries

Liangcheng Tan[1*], Zhisheng An[1], Chih-An Huh[2], Yanjun Cai[1], Chuan-Chou Shen[3], Liang-Jian Shiau[2], Libin Yan[1], Hai Cheng[4,5], R. Lawrence Edwards[5]

[1]State Key Laboratory of Loess and Quaternary Geology, Institute of Earth Environment, Chinese Academy of Sciences, Xi'an 710075, China

[2]Institute of Earth Sciences, Academia Sinica, Taipei 11529, Taiwan

[3]High-precision Mass Spectrometry and Environment Change Laboratory (HISPEC), Department of Geosciences, National Taiwan University, Taipei 106, Taiwan

[4] Institute of Global Environmental Change, Xi'an Jiaotong University, Xi'an 710049, China

[5] Department of Earth Sciences, University of Minnesota, Minneapolis 55455, USA

*To whom correspondence should be addressed. E-mail: tanlch@ieecas.cn

Supplementary Figures

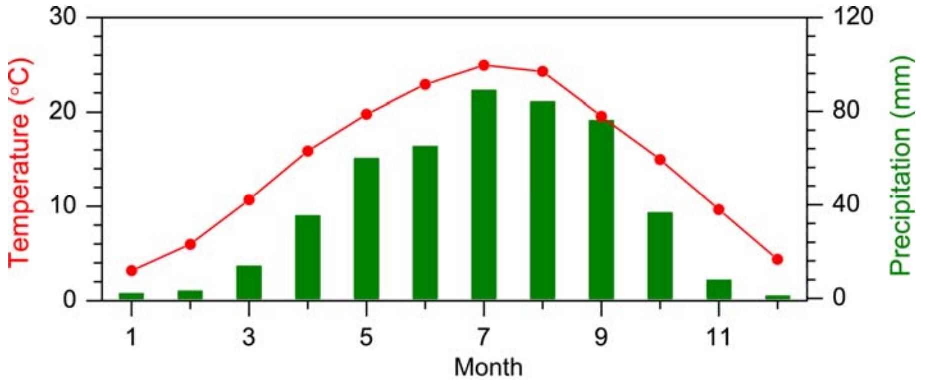

Fig. S1: Monthly rainfall (green) and temperature (red) at Wudu meteorological station 60 km southwest of Wayu Cave (data from 1951 to 2012 CE).

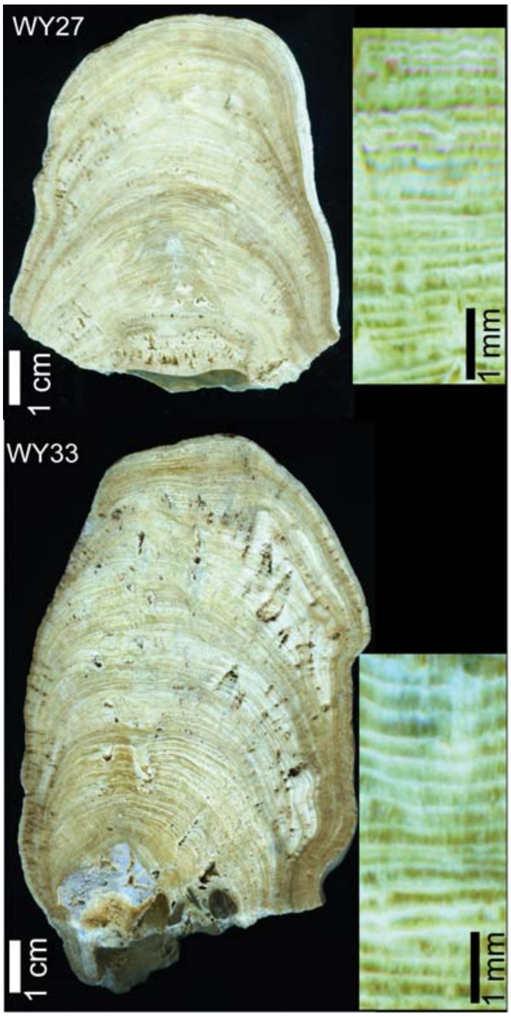

Fig. S2: Sections of stalagmite WY27 and WY33 showing well-developed annual laminae with alternating dark compact layers (DCL) and white porous layers (WPL).

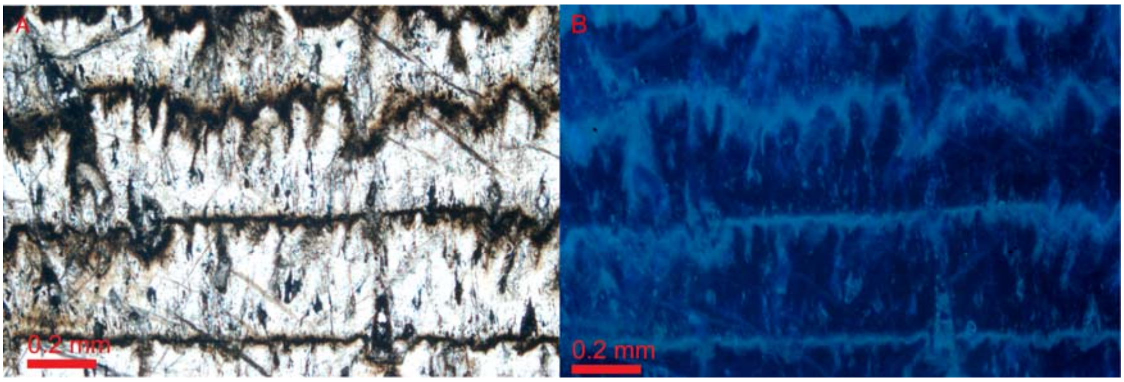

Fig. S3: The visible DCL-WPL annual layers of WY33 observed under (A) transmission light microscope and (B) UV light microscope. The visible white layers are opaque under transmission light microscope, but luminescent under mercury light source UV light microscope.

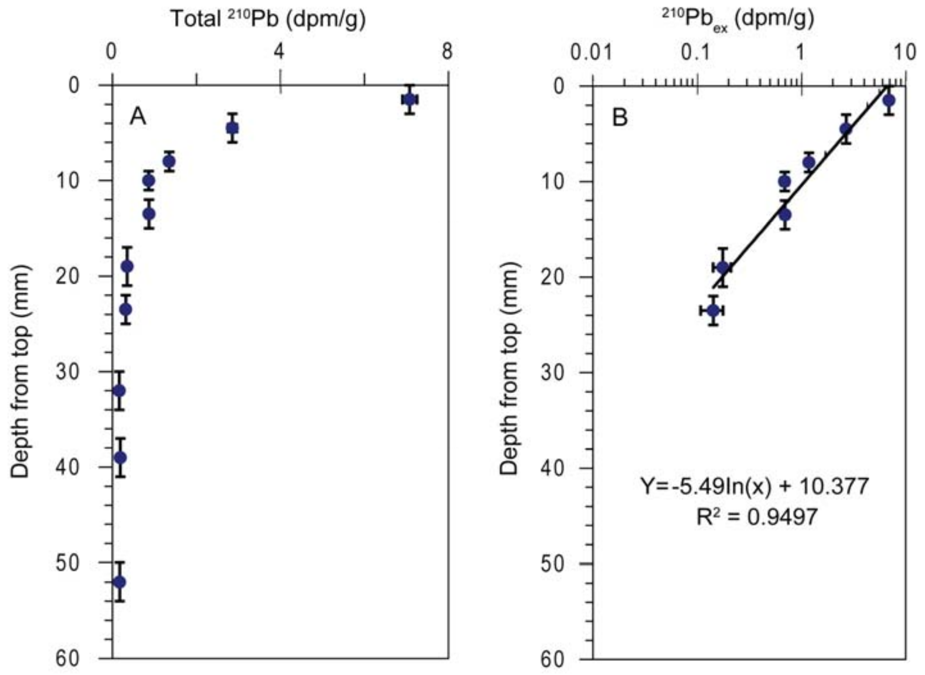

Fig. S4: Plots of (A) total ^{210}Pb activity and (B) excess ^{210}Pb activity ($^{210}Pb_{ex}$) versus depth in WY27. The supported ^{210}Pb activity level is 0.176 dpm/g, the average total ^{210}Pb activity at depths below 30 cm where excess ^{210}Pb is virtually nil. A mean growth rate of 0.171 mm/yr for the top 23.5 mm of WY27 is determined by fitting the excess ^{210}Pb activities (black line in panel B).

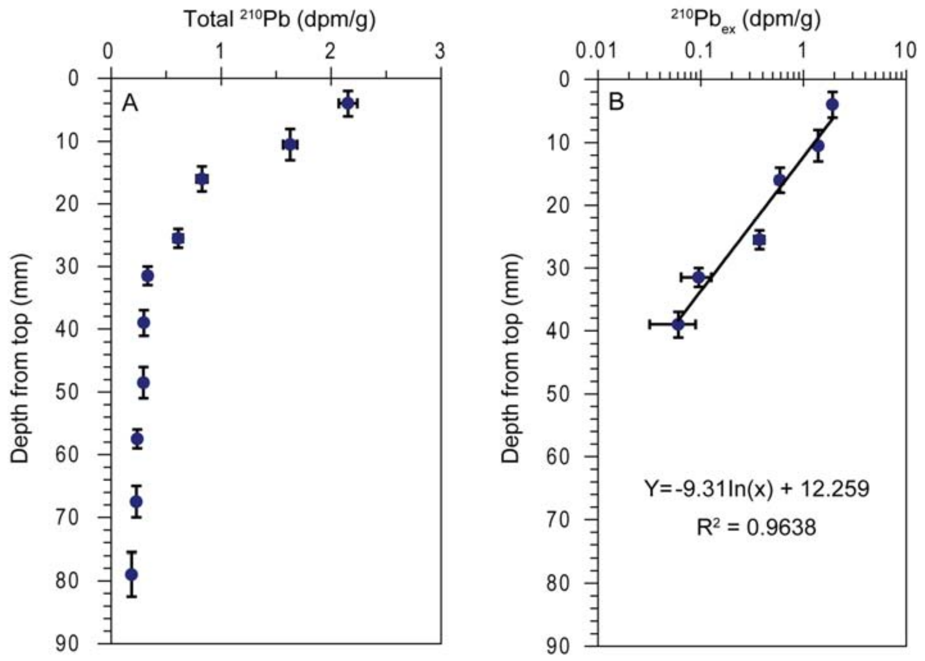

Fig. S5: Plots of (A) total ^{210}Pb activity and (B) excess ^{210}Pb activity ($^{210}Pb_{ex}$) versus depth in WY33. The supported ^{210}Pb activity level is 0.236 dpm/g. A mean growth rate of 0.289 mm/yr for the top 39 mm of WY33 is determined by fitting the excess ^{210}Pb activities (black line in panel B).

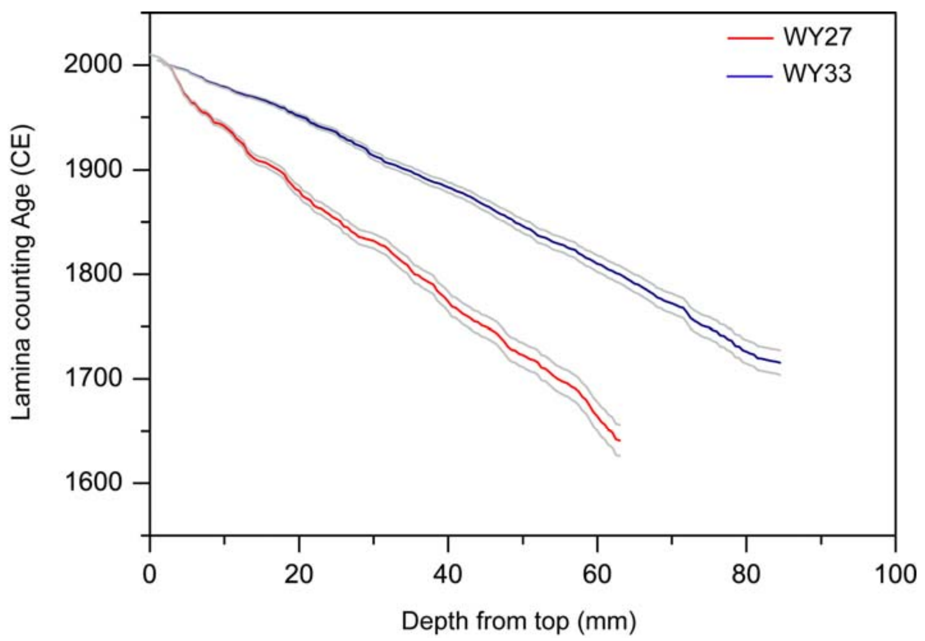

Fig. S6: Laminal chronologies of WY27 (red) and WY33 (green). The gray lines represent a ±4% error applied to the lamina counts of the two stalagmites. This error limits are based on the statistics of four times counting from two different paths by two persons.

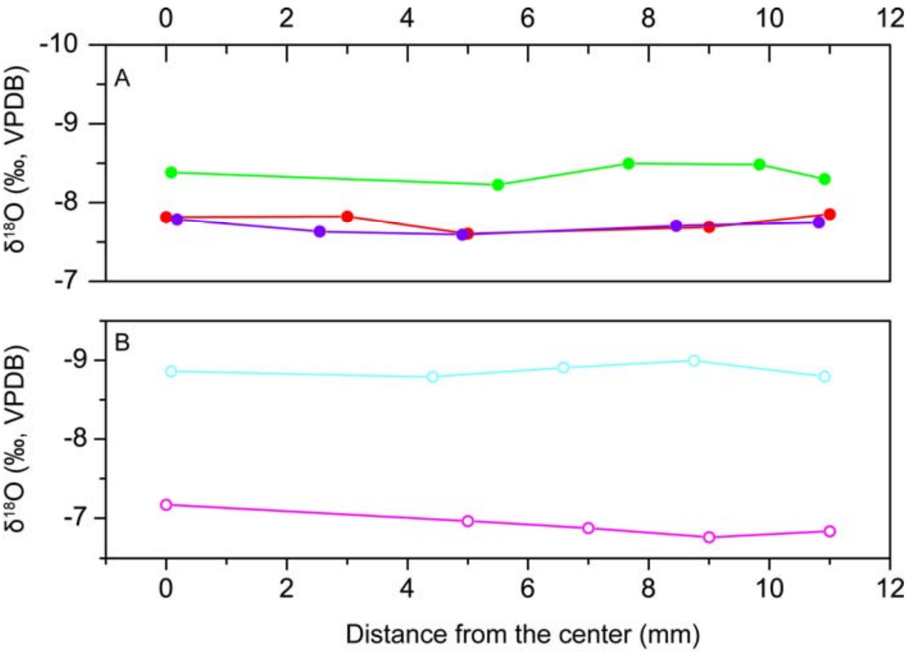

Fig. S7: "Hendy test"[18] on (A) three growth bands of stalagmite WY33 and (B) two growth bands of stalagmite WY27.

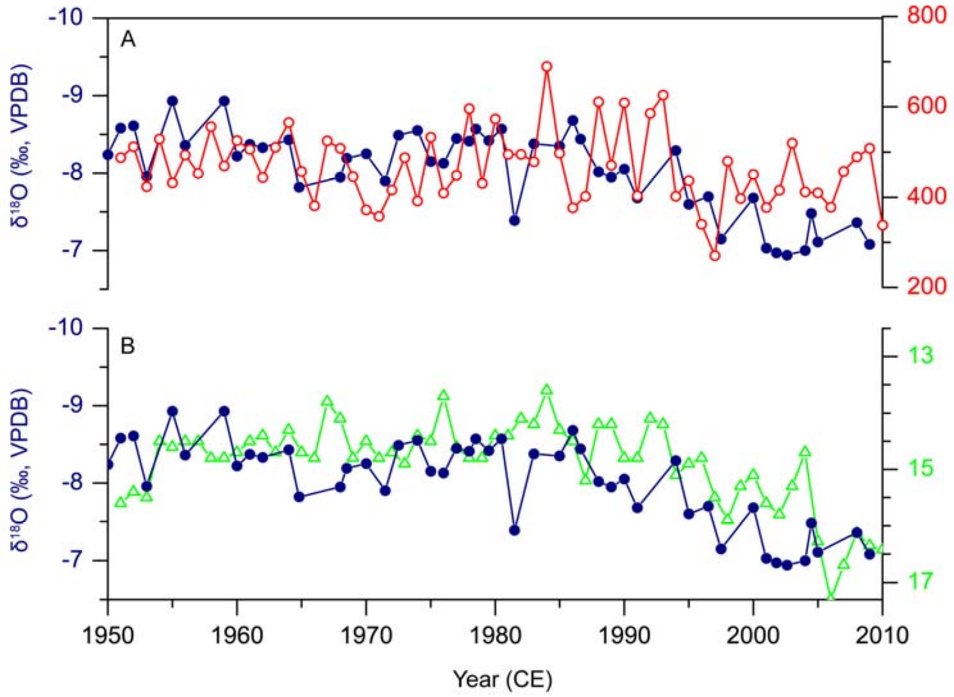

Fig. S8: Correlation between the WY33 $\delta^{18}O$ record (blue) and (A) local precipitation (red) and (B) temperature (green) recorded at Wudu meteorological station 60 km to the southwest of Wuya Cave during 1951-2009 CE. The WY33 $\delta^{18}O$ record presents a significant negative correlation to local precipitation record (r=-0.44) and a strong positive correlation (r= 0.68) with local temperature at the 0.01 level (To eliminate possible offsets in year-to-year correlations, 3-points running means are used for correlation analysis).

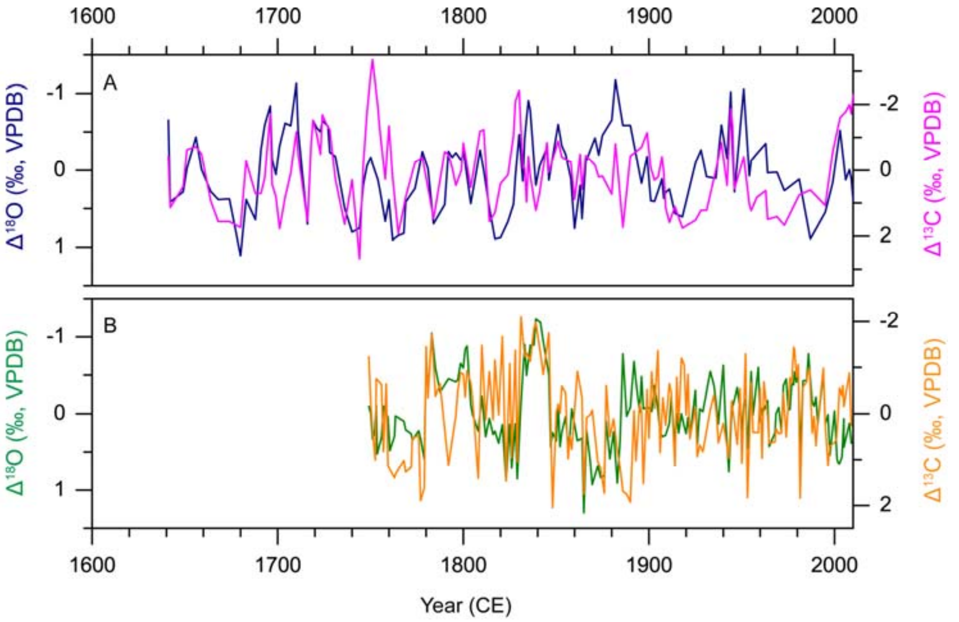

Fig. S9: Comparisons of detrended $\delta^{18}O$ and $\delta^{13}C$ records of (A) WY27 and (B) WY33

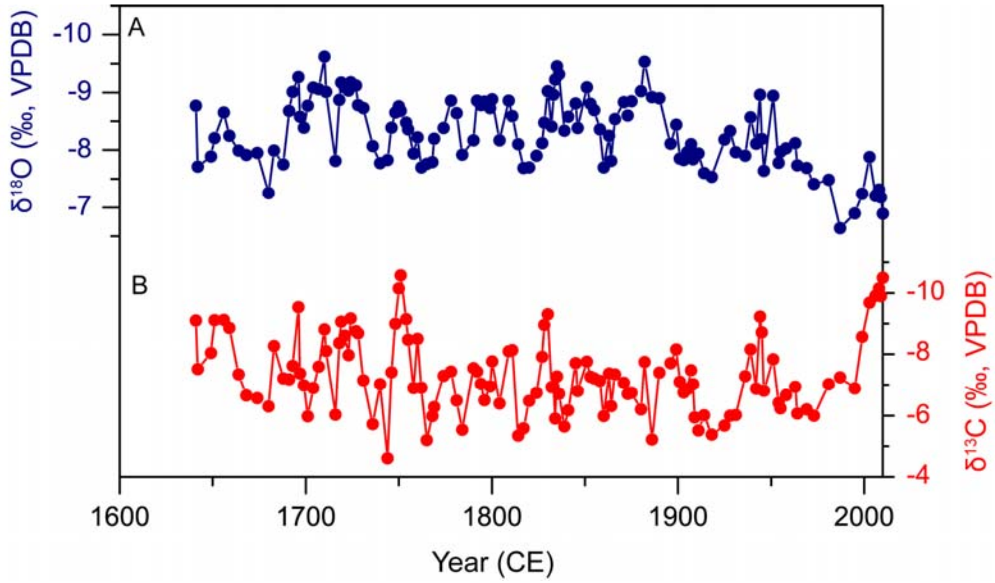

Fig. S10: The (A) $\delta^{18}O$ and (B) $\delta^{13}C$ records of WY27

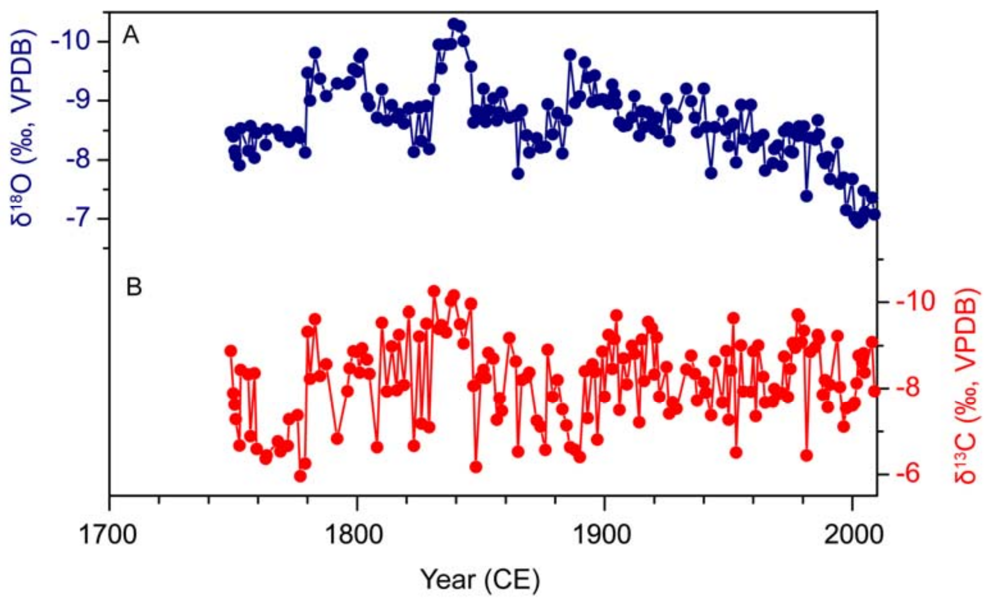

Fig. S11: The (A) $\delta^{18}O$ and (B) $\delta^{13}C$ records of WY33

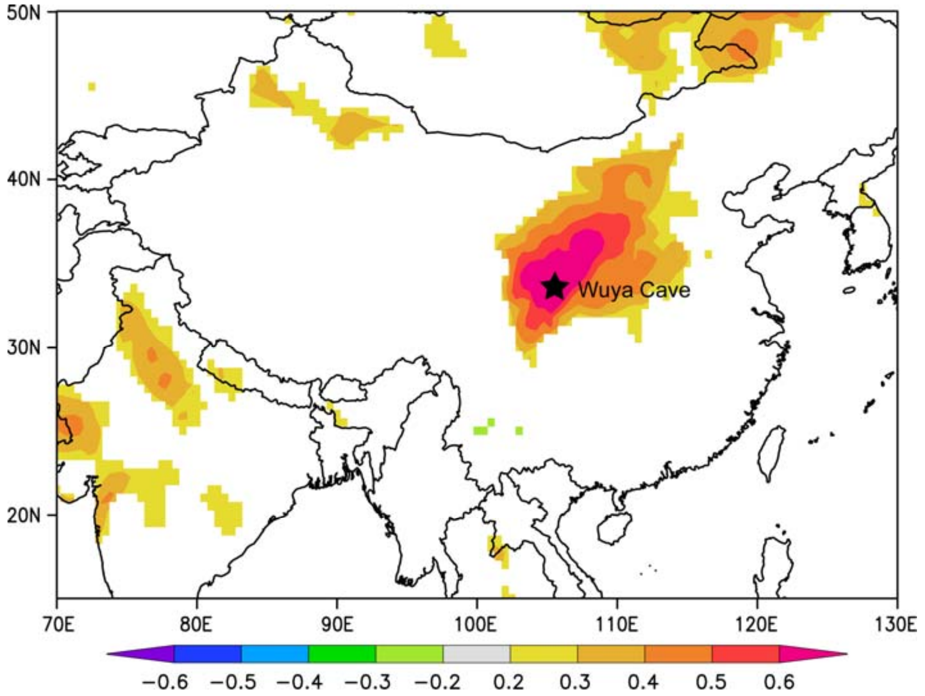

Fig. S12: Spatial correlation between annual precipitation in Wuya Cave area (105-105.5°E, 33.5-34°N) and the CRU TS3.21 precipitation grid datasets during 1950-2011 CE. The star indicates the location of Wuya Cave. The scale on the bottom shows the correlation coefficients represented by different colors. The analysis was performed by the KNMI Climate Explorer (*ref. S1*).

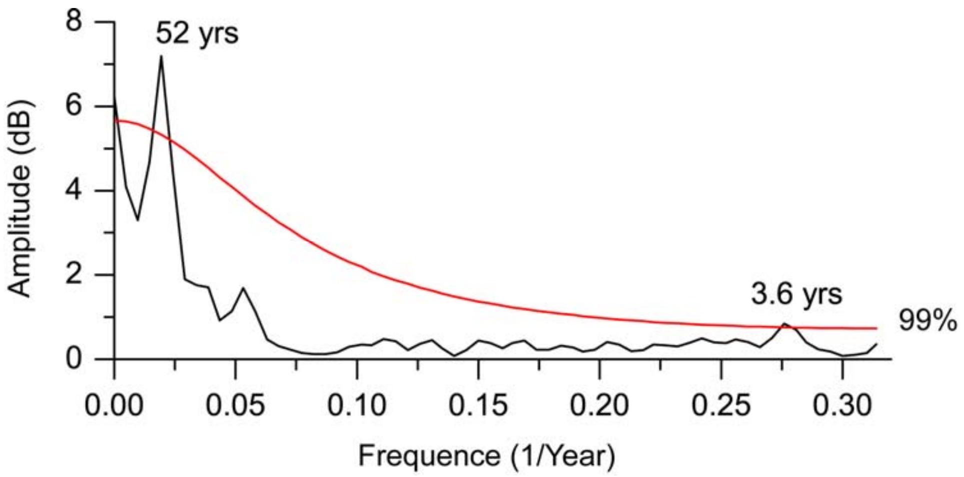

Fig. S13: Power spectrum analysis[58] of the WY33 $\delta^{18}O$ record. The periodicities of 52 yrs and 3.6 yrs are significant at the 99% false-alarm level.

Supplementary Tables

Table S1: U-Th data and ^{230}Th dates for stalagmite WY27 and WY33 from Wuya Cave.

Sample ID	Depth (mm)	^{238}U (ppb)	^{232}Th (ppt)	^{230}Th / ^{232}Th (atomic x10^{-6})	^{234}U (measured)	^{230}Th / ^{238}U (activity)	^{230}Th Age (yr) (uncorrected)	^{230}Th Age (yr) (corrected)	^{234}U$_{Initial}$ (corrected)	^{230}Th Age (yr BP) (corrected)
**WY27**										
**WY27-1**	**0.75**	720.2±2.0	7358±148	5±0	872.7±3.3	0.0030±0.0002	173±11	**14±113**	873±3	**-47±113**
**WY27-2**	**34.5**	396.0±1.3	11565±234	7±0	867.9±5.4	0.0121±0.0004	711±22	**255±323**	869±5	**194±323**
**WY27-3**	**55**	278.9±0.9	19251±389	6±0	841.2±6.0	0.0254±0.0006	1512±38	**417±776**	842±6	**356±776**
**WY33**										
**WY33-1**	**0.5**	563.0±2.5	25579±521	3±0	874.5±6.3	0.0093±0.0002	544±14	**-163±501**	874±6	**-224±501**
**WY33-2**	**30.5**	306.7±1.3	12517±255	5±0	867.1±8.3	0.0127±0.0005	747±30	**109±452**	867±8	**48±452**
**WY33-3**	**50.5**	272.8±0.9	16034±324	5±0	858.8±6.6	0.0179±0.0006	1054±39	**131±655**	859±7	**70±655**
**WY33-4**	**72**	357.0±1.2	27866±565	4±0	850.3±6.2	0.0201±0.0005	1190±31	**-43±874**	850±7	**-104±874**

The decay constant values used were: λ_{230} = 9.1705 x 10^{-6} yr^{-1}, λ_{234} = 2.8221 x 10^{-6} yr^{-1} (*ref. 55*) and λ_{238} = 1.55125 × 10^{-10} y^{-1}(*ref. S2*). Age corrections for all the subsamples were calculated using an estimated atomic ^{230}Th/^{232}Th ratio of 4.4 ± 2.2 ppm. Depths along the growth axis are relative to the top (youngest surface) of the stalagmites. Year BP: year before present (1950 CE).

Table S2. ^{210}Pb activity* measured in stalagmite WY27 and WY33

Depth from top (mm)	Total ^{210}Pb (dpm/g)			$^{210}Pb_{ex}$ (dpm/g)			Time of deposition (CE)		
WY27									
0–3	7.075	±	0.173	6.899	±	0.173	2002.4	±	8.8
3–6	2.857	±	0.104	2.681	±	0.105	1984.8	±	8.8
7–9	1.355	±	0.072	1.179	±	0.074	1964.3	±	5.9
9–11	0.866	±	0.054	0.690	±	0.055	1952.6	±	5.9
12–15	0.871	±	0.051	0.695	±	0.053	1932.1	±	8.8
17–21	0.352	±	0.032	0.176	±	0.034	1899.8	±	11.7
22–25	0.318	±	0.032	0.142	±	0.034	1873.5	±	8.8
30–34	0.165	±	0.022		-			-	
37–41	0.190	±	0.021		-			-	
50–54	0.174	±	0.020		-			-	
WY33									
2–6	2.153	±	0.084	1.916	±	0.095	1997.3	±	6.9
8–13	1.627	±	0.064	1.391	±	0.078	1974.9	±	8.6
14–18	0.825	±	0.048	0.589	±	0.066	1955.9	±	6.9
24–27	0.610	±	0.042	0.373	±	0.061	1923.1	±	5.2
30–33	0.332	±	0.031	0.096	±	0.054	1902.3	±	5.2
37–41	0.297	±	0.029	0.061	±	0.053	1876.4	±	6.9
46–51	0.293	±	0.028		-			-	
56–59	0.237	±	0.020		-			-	
65–70	0.229	±	0.021		-			-	
76–83	0.186	±	0.019		-			-	

* The listed ^{210}Pb activities are decay-corrected back to 1 March 2011 (*i.e.*, 2011.16 CE), the date of sample collection. Time of deposition of each sampling layer (T) is calculated by: $T = 2011.16 - Z/S$, where Z is depth (mm) of the sample layer and S (mm/yr) is the accumulation rate of the stalagmite specimen derived from the depth profile of excess ^{210}Pb ($^{210}Pb_{ex}$), as shown in Figures S4 and S5.

Table S3. Oxygen isotopic data of stalagmites WY33 and WY27 from Wuya Cave. Depths are relative to the top (youngest surface) of the stalagmites and are measured along the growth axis. Ages are determined by annual layer counting. Oxygen isotope ratios are expressed in δ notation, and the per mil values are derived with respect to the Vienna Pee-Dee Belemnite standard.

Depth (mm)	Age (CE)	$\delta^{18}O$ (VPDB, ‰)	Depth (mm)	Age (CE)	$\delta^{18}O$ (VPDB, ‰)	Depth (mm)	Age (CE)	$\delta^{18}O$ (VPDB, ‰)	Depth (mm)	Age (CE)	$\delta^{18}O$ (VPDB, ‰)
WY33											
1	2009	-7.08	16.5	1972	-7.90	32	1921	-8.72	47.5	1876	-8.23
1.5	2008	-7.36	17	1970	-8.25	32.5	1920	-8.52	48	1874	-8.21
2	2005	-7.11	17.5	1969	-8.19	33	1919	-8.62	48.5	1873	-8.37
2.5	2004.5	-7.48	18	1968	-7.95	33.5	1918	-8.80	49	1870	-8.13
3	2004	-7.00	18.5	1965	-7.82	34	1916	-8.56	49.5	1869	-8.42
3.5	2003	-6.94	19	1964	-8.43	34.5	1915	-8.82	50	1867	-8.84
4	2002	-6.97	19.5	1962	-8.33	35	1914	-8.41	50.5	1865	-7.77
4.5	2001	-7.03	20	1961	-8.37	35.5	1912	-9.08	51	1864	-8.75
5	2000	-7.68	20.5	1960	-8.22	36	1911	-8.73	51.5	1862	-8.72
5.5	1998	-7.15	21	1959	-8.93	36.5	1909	-8.59	52	1859	-9.14
6	1997	-7.70	21.5	1956	-8.36	37	1908	-8.58	52.5	1858	-8.80
6.5	1995	-7.60	22	1955	-8.93	37.5	1906	-8.64	53	1857	-8.67
7	1994	-8.29	22.5	1953	-7.96	38	1905	-8.95	53.5	1855	-9.04
7.5	1991	-7.68	23	1952	-8.61	38.5	1904	-9.11	54	1853	-8.84
8	1990	-8.05	23.5	1951	-8.58	39	1903	-9.27	54.5	1852	-8.65
8.5	1989	-7.95	24	1950	-8.24	39.5	1902	-8.95	55	1851	-9.20
9	1988	-8.02	24.5	1949	-8.51	40	1900	-9.01	55.5	1850	-8.72
9.5	1987	-8.44	25	1948	-8.82	40.5	1899	-9.02	56	1848	-8.83
10	1986	-8.68	25.5	1945	-8.56	41	1897	-9.01	56.5	1847	-8.64
10.5	1985	-8.35	26	1943	-7.78	41.5	1896	-9.43	57	1846	-9.58
11	1983	-8.38	26.5	1941	-8.56	42	1895	-8.98	57.5	1843	-10.01
11.5	1982	-7.39	27	1940	-9.20	42.5	1893	-9.39	58	1842	-10.26
12	1981	-8.57	27.5	1937	-8.47	43	1892	-9.65	58.5	1839	-10.30
12.5	1980	-8.42	28	1936	-8.72	43.5	1890	-9.07	59	1838	-9.96
13	1979	-8.57	28.5	1935	-8.99	44	1888	-8.96	59.5	1836	-9.95
13.5	1978	-8.41	29	1933	-9.20	44.5	1886	-9.78	60	1834	-9.55
14	1977	-8.45	29.5	1929	-8.72	45	1885	-8.67	60.5	1833	-9.95
14.5	1976	-8.13	30	1927	-8.77	45.5	1883	-8.11	61	1831	-9.19
15	1975	-8.15	30.5	1926	-8.32	46	1881	-8.79	61.5	1829	-8.19
15.5	1974	-8.55	31	1925	-9.03	46.5	1879	-8.44	62	1828	-8.91
16	1973	-8.49	31.5	1922	-8.46	47	1877	-8.94	62.5	1826	-8.32

Continue to next page

Table S3 (Cont.)

Depth (mm)	Age (CE)	$\delta^{18}O$ (VPDB, ‰)	Depth (mm)	Age (CE)	$\delta^{18}O$ (VPDB, ‰)	Depth (mm)	Age (CE)	$\delta^{18}O$ (VPDB, ‰)	Depth (mm)	Age (CE)	$\delta^{18}O$ (VPDB, ‰)
63	1825	-8.89	81.5	1753	-8.54	15	1908	-7.84	33.5	1814	-8.10
63.5	1823	-8.14	82	1752.5	-7.91	15.5	1907	-8.10	34	1811	-8.59
64	1821	-8.87	83	1751	-8.07	16	1905	-7.96	34.5	1809	-8.86
64.5	1819	-8.62	83.5	1750.5	-8.16	16.5	1903	-7.83	35	1804	-8.17
65	1817	-8.77	84	1750	-8.40	17	1901	-7.85	35.5	1800	-8.88
65.5	1816	-8.72	84.5	1749	-8.47	17.5	1899	-8.44	36	1799	-8.73
66	1814	-8.92	**WY27**			18	1896	-8.11	36.5	1796	-8.84
66.5	1812	-8.67	0.1	2010	-6.89	18.5	1890	-8.90	37	1794	-8.78
67	1810	-9.19	0.5	2009	-7.18	19	1886	-8.92	37.5	1792	-8.86
67.5	1808	-8.72	1	2008	-7.31	19.5	1882	-9.53	38	1790	-8.17
68	1805	-8.92	1.5	2006	-7.20	20	1880	-9.02	38.5	1784	-7.92
68.5	1804	-9.04	2	2003	-7.88	20.5	1875	-8.85	39	1781	-8.64
69	1802	-9.79	2.5	1999	-7.24	21	1873	-8.60	39.5	1778	-8.86
69.5	1801	-9.74	3	1995	-6.90	21.5	1871	-8.83	40	1774	-8.38
70	1800	-9.49	3.5	1987	-6.64	22	1866	-8.54	40.5	1769	-8.20
70.5	1799	-9.54	4	1981	-7.48	22.5	1864	-7.81	41	1768	-7.79
71	1797	-9.31	4.5	1973	-7.41	23	1863	-8.25	41.5	1765	-7.76
71.5	1796	-9.28	5	1969	-7.69	23.5	1860	-7.70	42	1762	-7.70
72	1792	-9.29	5.5	1964	-7.73	24	1858	-8.36	42.5	1760	-8.22
72.5	1788	-9.08	6	1963	-8.12	24.5	1855	-8.69	43	1758	-7.94
73	1785	-9.37	6.5	1958	-8.03	25	1853	-8.80	43.5	1755	-8.36
73.5	1783	-9.81	7	1955	-7.97	25.5	1851	-9.09	44	1754	-8.48
74	1781	-9.00	7.5	1954	-7.78	26	1846	-8.38	44.5	1751	-8.68
74.5	1780	-9.47	8	1951	-8.94	26.5	1845	-8.81	45	1750	-8.76
75	1779	-8.13	8.5	1946	-7.64	27	1841	-8.58	45.5	1748	-8.65
75.5	1777	-8.38	9	1945	-8.19	27.5	1839	-8.34	46	1746	-8.39
76	1776	-8.47	9.5	1944	-8.96	28	1836	-9.32	46.5	1744	-7.83
76.5	1773	-8.31	10	1942	-8.11	28.5	1835	-9.45	47	1740	-7.77
77	1772	-8.39	10.5	1939	-8.57	29	1834	-9.22	47.5	1736	-8.07
77.5	1769	-8.41	11	1936	-7.90	29.5	1833	-8.96	48	1731	-8.73
78	1768	-8.51	11.5	1931	-7.96	30	1832	-8.41	48.5	1728	-8.78
78.5	1764	-8.53	12	1928	-8.33	30.5	1830	-9.02	49	1727	-9.12
79	1763	-8.26	12.5	1925	-8.18	31	1828	-8.48	49.5	1724	-9.18
79.5	1760	-8.46	13	1918	-7.53	31.5	1827	-8.12	50	1723	-9.03
80	1759	-8.04	13.5	1914	-7.60	32	1824	-7.90	50.5	1721	-9.08
80.5	1757	-8.58	14	1911	-7.94	32.5	1820	-7.70	51	1719	-9.17
81	1756	-8.16	14.5	1909	-7.91	33	1817	-7.69	51.5	1718	-8.87

Continue to next page

Table S3 (Cont.)

Depth (mm)	Age (CE)	$\delta^{18}O$ (VPDB, ‰)	Depth (mm)	Age (CE)	$\delta^{18}O$ (VPDB, ‰)	Depth (mm)	Age (CE)	$\delta^{18}O$ (VPDB, ‰)	Depth (mm)	Age (CE)	$\delta^{18}O$ (VPDB, ‰)
52	1716	-7.81	55	1699	-8.39	58	1683	-7.99	61	1656	-8.65
52.5	1711	-9.01	55.5	1697	-8.58	58.5	1680	-7.25	61.5	1651	-8.21
53	1710	-9.62	56	1696	-9.27	59	1674	-7.95	62	1649	-7.89
53.5	1707	-9.06	56.5	1693	-9.01	59.5	1668	-7.91	62.5	1642	-7.71
54	1704	-9.08	57	1691	-8.68	60	1664	-7.99	63	1641	-8.77
54.5	1701	-8.77	57.5	1688	-7.75	60.5	1659	-8.25			

References for Supplementary Materials:

S1. Van Oldenborgh, G. J. & Burgers, G. Searching for decadal variations in ENSO precipitation teleconnections. *Geophys. Res. Lett.* **32,** *L15701* (2005).

S2. Jaffey, A. H. K., Flynn, F., Glendenin, L. E., Bentley, W. C. & Essling, A. M. Precision measurement of half-lives and specific activities of ^{235}U and ^{238}U. *Physics Reviews C* **4**, 1889-1906 (1971).
